# A qualitative analysis of stress experiences and coping strategies in adolescents with Crohn's disease

**DOI:** 10.1186/s12888-023-05241-6

**Published:** 2023-10-20

**Authors:** Xiao-juan Zhou, Shuai Huang

**Affiliations:** Department of General Surgery, Xinhua Hospital Affiliated to Shanghai Jiao Tong University School of Medicine, 1665 Kangjiang Road, Yangpu District, Shanghai, 200082 China

**Keywords:** Adolescents, Coping styles, Crohn’s disease, Inflammatory bowel disease, Stress

## Abstract

**Background:**

In this study, we investigated the coping mechanisms and stress perceptions of adolescent patients with Crohn's disease.

**Methods:**

Using semi-structured face-to-face interviews, we conducted an extensive qualitative study of the disease perceptions, stress experiences, and corresponding coping mechanisms in adolescent patients with Crohn's disease. We used Colaizzi content analysis to synthesize the themes.

**Results:**

The two main themes in this study were inappropriate coping mechanisms and physical and psychological stress. The primary initiators of physical and psychological stress in adolescents with Crohn's disease were weak disease perception, symptom distress, negative emotions, lack of support, and multiple stressors. The decrease in self-management and self-control induced by the initiators led to changes in cognition, emotions, and attitudes, which subsequently led to poor coping behavior.

**Conclusion:**

Adolescents with Crohn's disease can better combat the condition by implementing appropriate coping strategies. Their mental health should be given attention, and a multidisciplinary team should be assembled to provide them with supportive care.

## Background

Granulomas are the hallmark of Crohn's disease (CD), a chronic inflammatory disease of the digestive tract. The terminal ileum and adjacent areas of the colon are most commonly impacted [1]. Although the precise etiology and mechanisms of CD are not well understood, they probably involve immunological responses, environmental triggers, and genetic factors. Abdominal pain, diarrhea, and bloody stools are typical symptoms. According to recent data from China, CD is on the rise, with an estimated incidence of 1.4 cases per 100,000 persons, translating to 27,000 to 43,000 new cases every year [2].

Due to their youth and ongoing physical and emotional development, adolescents with Crohn's disease who are diagnosed between the ages of 13 and 18 confront particular difficulties [3]. When attempting to balance the responsibilities of both controlling their disease and their academics, they frequently choose less effective or unhealthy coping mechanisms. Stress has the ability to exacerbate the progression of the disease by lowering the pain threshold in the intestines and interfering with the protective role of the intestinal lining. Additionally, stress-related negative emotions can hinder the healing process. As a result, adolescents with Crohn's disease need more intensive mental health support. Surprisingly, there is a lack of research on how Crohn's disease in adolescents affects their ability to manage stress [4]. We carried out a qualitative study to improve clinical procedures and the management of this disease in adolescent patients. Semi-structured interviews were used in this study to better understand how adolescents with Crohn's disease interpret stress and their coping mechanisms. Our goal was to find themes and analyze the influencing factors [5].

## Methods

### Study participants and methods

During the period from October 2021 to January 2022, we selected adolescent patients diagnosed with Crohn's disease who had sought medical care at a hospital associated with Shanghai Jiao Tong University School of Medicine. We utilized a combination of theoretical and purposive sampling methods to identify participants, followed by conducting face-to-face semi-structured interviews. The research adhered to the qualitative research principle of thematic saturation, meaning that we concluded data analysis when no additional novel information surfaced. Ultimately, our study included a total of 12 adolescents diagnosed with Crohn's disease.

#### Inclusion criteria

1. Conform to the diagnostic requirements for Crohn's disease [6].
2. The age was within the adolescent age group of 13–18 years but did not exceed 18 years [3].
3. Voluntary participation after informed consent.
4. Able to communicate and comprehend in relation to the questionnaires.

#### Exclusion criteria

1. Decline to take part in this research and displaying reluctance to participate.
2. Patients experiencing language communication disorders, inability to comprehend or complete the questionnaires.
3. Patients grappling with severe medical conditions, such as significant cardiovascular and cerebrovascular ailments, as well as malignant tumors.
4. Patients with mental disorders.
5. Patients complicated with other gastrointestinal diseases.

### Outline of interview

Prior to the formal interview, our team would typically engage in dialogues and discussions with the study participants to gauge their perspectives, attitudes, and levels of stress. Additionally, we developed an initial interview strategy by consulting previously published work, theoretical analysis, and professional advice. We also conducted preliminary interviews with two participants (their responses were excluded from the final interview analysis). The preliminary interviews provided valuable information that was used to modify and improve the original interview strategy, which eventually resulted in the formal interview outline being created after expert deliberation.

#### Interview outline:

1. What is your level of knowledge regarding Crohn's disease, and what were your initial feelings upon receiving a Crohn's disease diagnosis?
2. Can you describe the journey that you have taken from the time of diagnosis to the present day?
3. In what ways has this condition affected your life and academic pursuits, and which factors have played the most significant role?
4. How did you cope with the consequences of the condition?
5. What are the feelings of your friends and family towards your condition, and what kind of treatment or support do you prefer from others?
6. What are your expectations regarding the progression of your Crohn's disease?

### Conducting the interview

Two investigators conducted interviews in a small, serene meeting room, and the entire process was recorded. Each interview took approximately 30 to 40 min, during which the investigators paid close attention to non-verbal cues, facial expressions, and other relevant characteristics to gain a more profound insight into the mental states of the participants. After completing each interview, the recordings were transcribed. To analyze the collected data, we employed the Colaizzi technique of content analysis, which involves seven key steps [7]. These steps include:

1. Thoroughly reviewing the interview data to develop a general understanding of its content.
2. Identifying and extracting meaningful statements from the data.
3. Summarizing and extracting meaningful statements, then encoding them for analysis.
4. Summarizing the encoded statements and identifying common concepts to create themes or thematic groups.
5. Integrating the results to construct a comprehensive overall description.
6. Using the statements as the foundational framework for understanding the phenomenon.
7. Finally, the research findings were shared with the participants to validate the accuracy and authenticity of the content.

## Results

### General data characteristics of adolescent patients with Crohn’s disease

Twelve adolescents with Crohn's disease between the ages of 13 and 18 participated in qualitative interviews for this study; seven had finished junior high school and five had finished high school; seven had complications; seven received medication; two had been taking long-term oral medication; and two were being fed nasally. The 12 respondents were given the sequential numbers P1, P2, P3, P4, P5, P6, P7, P8, P9, P10, P11, and P12. The data analysis ultimately produced the following themes (see Table 1).

**Table 1:** General information of adolescent patients with Crohn's disease

Serial number	Age	Gender	Learning status	Residential status	Medical insurance	Course of disease	Complications	Medication
P1	16	Male	Ninth grade	Living with parents	Yes	2	Anal fistula	No
P2	17	Male	Senior one	Living with parents	Yes	4	Perianal abscess	Regular infliximab injections
P3	14	Male	Eighth grade	Boarding school	None (foreigner)	2	None	Regular infliximab injections
P4	15	Male	Ninth grade	Living with mother	None (foreigner)	3	Anal fistula	Oral medication
P5	13	Female	Seventh grade	Living with parents	None (foreigner)	6	None	Regular infliximab injections
P6	15	Male	Ninth grade	Living with father	Yes	1	None	No
P7	15	Female	Ninth grade	Boarding school	Yes	4	None	Regular infliximab injections
P8	16	Male	Senior one	Living with parents	Yes	5	Anal fistula	Regular infliximab injections
P9	17	Male	Senior one	Living with grandmother	None (foreigner)	4	Anal fistula	No
P10	15	Female	Ninth grade	Living with parents	Yes	3	None	Oral medication
P11	17	Female	Senior two	Living with mother	None (foreigner)	6	Perianal abscess	Regular infliximab injections
P12	16	Male	Senior one	Living with parents	Yes	4	Anal fistula	Regular infliximab injections

### Main causes of physical and mental stress

#### Inadequate cognition

Uncertainty is likely to develop due to a lack of knowledge about the disease. It is impossible to predict disease progression, probable relapses, and prognosis when there is a lack of appropriate understanding about the disease. This ignorance can easily result in increased stress levels, which eventually lowers the patient's quality of life as a whole and lessens the therapeutic benefit [8, 9].

Some patients claimed that the absence of expert advice from healthcare experts is to blame for their incomplete cognition of the disease. The current healthcare system frequently falls short of providing the required assistance for learning about and being aware of the disease. The adolescent patients, who have a long journey ahead of them, are left feeling confused and uncertain as a result.

P3: "I find many aspects confusing, like what to eat. Typically, I just follow my mother's instructions for meals. There are foods I really want to try, but I'm uncertain if I can. I'm filled with frustration and anger, and it's all because of my condition; I have no idea what the future holds for me."

#### Symptom distress

The main difficulties that patients seem to encounter are symptoms related to the illness, such as diarrhea, stomach pain, and the occurrence of a concomitant anal fistula. As an illustration, a key symptom is the incessant need to use the restroom at all times and locations [10]. Adolescents who experience these disease-related symptoms experience varied degrees of emotional discomfort, experience constant stress and need specialist care.

P4: "I frequently find myself needing to visit the restroom. During class, I often feel compelled to go, and it's embarrassing because I sense that my classmates are giving me odd looks whenever I do."

Even after undergoing surgery for the anal fistula, individuals with a concurrent anal fistula still experience anal discharge, which can result in considerable psychological distress.

P8: "There is often a discharge from the incision of the anal fistula, but I feel too ashamed to discuss it with my parents, so I occasionally use tissue paper to hide it. The discomfort has been so intense that it has caused me to lose sleep on several occasions."

#### 2.2.3 Negative emotions

Negative emotions in adolescent patients with Crohn's disease, such as anxiety and embarrassment, are signs of the stress they go through at this crucial time in their development known as puberty. The symptoms of the condition lead to ongoing stress and negative feelings. In our study, some participants expressed their anxiety about the disease's uncertainty, feelings of shame about their self-image, guilt toward their families for having to take them to so many doctor's appointments, and lower self-esteem because their peers were uncomfortable with things like the unpleasant restroom odors caused by their frequent trips to the bathroom.

P1: "Whenever I notice blood in my stool, I become apprehensive, fearing the severity of my condition and unsure about my life expectancy (accompanied by an awkward smile). My mother keeps taking me to the doctor, and at times, she appears quite irritated, complaining about the added responsibilities. Other kids are healthy, and I feel a sense of guilt and a desire to apologize."

P2: "Because of my illness, numerous hospital stays, and injections, my father decided to switch me to a different school. Although it was more convenient to study from home, I worried that I wouldn't be as effective in my studies and wouldn't adequately prepare for next year's senior entrance exam. I took a break from school with hopes of returning once my health improved, so I could prepare for the entrance exam at school. This decision distanced me from my peers, and I don't even have a graduation photo."

#### Lack of support

Numerous psychological and external factors, as well as others, can cause stress. These sources comprise elements like the influence of one's environment, other people's perceptions, and the state of the economy. Adolescents struggle to comprehend their condition at the same time, and if their families and medical experts are unable to provide them with enough assistance, they may turn to harmful coping strategies.

P12: "Every time I experience symptoms, I tend to forget specific advice—sometimes it's my mother warning against certain foods, other times it's my father advising against something else, and occasionally, at school, I'm unsure about what's safe to eat."

P6: "I feel a bit uneasy in large groups because I worry that my classmates might hear about my frequent restroom visits. I'm not sure how many public restrooms are available or how convenient they are."

It is obvious that patients' attitudes and emotions are substantially impacted by the lack of professional and emotional support, which in turn affects how they behave. Adolescents with Crohn's disease frequently find it difficult and prefer to keep their condition a secret from others. They worry about being regarded as different, are fearful of how their surroundings might change them, and fear not being understood.

#### Interaction of multiple pressures

Many adolescents dealing with Crohn's disease find themselves navigating the challenging terrain of middle and high school, which are pivotal phases in their education. Balancing the demands of learning with the burden of illness places a significant strain on these young patients, impeding their recovery and heightening the likelihood of relapse.

P3: "Undoubtedly, the greatest impact is on my academics, and frequent hospital visits eat away at my precious time. I constantly have to request time off, leaving me unable to catch up on missed classes. It's quite disheartening, and I fear my classmates may outperform me in the upcoming monthly exam. Sometimes, I struggle to fall asleep until the early morning hours.

P4: "Perhaps it's somewhat expected that I feel the pressure, but I've grown somewhat desensitized to it. My father takes me to the hospital, and I accompany him willingly. The same goes for medical tests. In the end, it doesn't matter; perhaps everyone experiences illness in a similar way."

### Inappropriate coping styles

Emotion-focused stress coping has been found to be more successful in reducing stress and improving the overall quality of life of people with Crohn's disease. Coping is crucial in helping people with Crohn's disease manage issues brought on by stress [11]. According to the theory of stress coping, three main coping techniques—emotion-focused coping, problem-focused coping, and meaning-focused coping—emerge. These techniques are selected depending on an evaluation of the particular situation at hand [12, 13]. While some patients actively use a variety of coping mechanisms to manage perceived stress, others may fall back on ineffective coping mechanisms, adopt unfavorable attitudes, look for ways to escape, or simply accept their condition passively.

#### Inadequate disease knowledge and mental exhaustion drive patients to make poor choices and react emotionally rather than rationally

Lack of adequate understanding of the disease and cognitive limitations can lead to inappropriate responses from patients, such as blindly seeking various treatments. This insufficient knowledge also increases uncertainty and makes patients and their families more susceptible to anxiety and tension, which can result in impulsive decisions and passive reactions [14].

P11: "I visited multiple hospitals and even consulted a Chinese medicine practitioner and tried Chinese remedies, but I didn't experience clear improvement, and my stomach pain persisted, ultimately leading me to seek help at a hospital."

P9: "I wasn't aware of why my father constantly advised me against consuming certain foods or beverages due to my illness. He consistently encouraged me to manage my diet, claiming that it would reduce my need for frequent hospitalizations, which made it challenging."

The insufficient knowledge of the disease significantly influences individuals' attitudes and subsequent behavior. Therefore, it is essential not only for patients and their caregivers to gain a comprehensive understanding of Crohn's disease but also for healthcare professionals to offer accurate guidance and actively disseminate knowledge about the condition to their patients [15].

#### Poor emotional control leads to negative emotions and defiant behavior

Individuals need to resist the urge to react emotionally to negative stimuli in social situations and when dealing with illness. However, poor emotional control can easily lead to negative emotions or even a loss of emotional regulation, creating a harmful cycle. Even when in remission from their illness, adolescents with Crohn's disease often experience anxiety and uncertainty. Hospitalization can further exacerbate emotional struggles for both patients and their caregivers [16].

P7: “I've visited several hospitals and consulted various specialists. Some of them have mentioned the need for additional test results. At times, I question whether it's truly Crohn's disease. I'm still young, and the thought of it saddens me.”

P3: “My mood is primarily influenced by my mother. Because of my illness, I switched to a school closer to home so I could stay at home. My frequent hospitalizations occasionally caused my mother to become upset. I feel a great deal of sensitivity, anxiety, and guilt. There were instances when I had diarrhea, but I didn't inform her because I didn't want to burden her or go to the hospital.”

Adolescents are in a critical phase of physical and psychological development, making them susceptible to rebellious thoughts. Without proper guidance, they may engage in rebellious behavior due to their vulnerability to negative emotions and disorders.

#### Reduction in self-management leads to negative attitudes and burnout behavior

Prolonged illness in individuals with Crohn's disease can lead to a depletion of their ability to manage and control their condition, resulting in negative feelings towards the disease, mental fatigue, and the adoption of burnout coping strategies [17, 18].

P10: "I've been unwell for five years, undergone numerous tests, and had several hospitalizations. I've been on medication for an extended period, which restricts my diet significantly. There are times when I really don't feel like taking my medication, and I occasionally miss doses. Despite not being allowed to have milk, I secretly indulge in it."

P5: "I diligently follow my doctor's instructions and do so under my parents' supervision. However, when I'm at school, I become quite apathetic and avoid taking my medication because I'm embarrassed about my classmates witnessing it."

Effective self-management of the disease plays a pivotal role in maintaining remission among patients with chronic conditions [19], but adolescents with chronic Crohn's disease, despite maintaining a positive coping attitude, often experience exhaustion.

#### Internal and external environmental factors lead to lack of support and inefficient coping

Research has shown that strong social support fosters positive interpersonal relationships and a positive attitude, which in turn leads to a favorable response to experiences, and vice versa [20]. Adolescents with Crohn's disease who have long-term illnesses and face negative attitudes from their family and friends experience a lack of social support and reduced social engagement. These unfavorable attitudes among patients lead to ineffective coping behavior [21, 22].

P5: "When I lived in a dormitory, my frequent bathroom trips upset my roommates due to the odor in the bathroom. This made me feel embarrassed, and for a while, I avoided going to school. My neighbor’s grandmother noticed that I was frequently at home and questioned me as to why I was not going to school. Now, I don't even want to go out, and I don't want to see my parents since they will keep questioning me about these issues."

P9: "Both of my parents work out of town, but they occasionally return to take me to the hospital. This time, the stool test results were not favorable. I eat whatever my grandmother prepares and the lunch in the school cafeteria."

In summary, a lack of support leads to decreased social connections, often resulting in patients and caregivers struggling to employ effective coping strategies, ultimately resulting in inadequate coping.

## Discussion

### Focus on mental health and individualized instructional measures

The results of the study show that adolescents with Crohn's disease experience a wide range of emotions, including hope and fear. They experience heightened worry and a decreased feeling of self-worth as a result of their numerous medical appointments and other healthcare-related difficulties. These adolescents consequently experience psychological trauma as a result of their low self-esteem and mood issues. According to a study, such psychological issues may play a role in the onset of Crohn's disease and need to be seriously considered [23].

Based on the results of our study, it is clear that adolescents with Crohn's disease experience a conflicting mood that is defined by both hope and anxiety. Their ongoing medical appointments and the accompanying costs only make their worry and low self-esteem problems worse, which leads to psychological anguish as a result of their emotional disorders and low self-esteem [24]. A study by Zhao found that patients with inflammatory bowel disease (IBD), including Crohn's disease, frequently experience a high prevalence of psychological symptoms, with anxiety accounting for a significant proportion (51.3%). Some respondents even reported experiencing insomnia as a result of anxiety brought on by stress [25]. This emphasizes how crucial it is to attend to the mental health issues of people with Crohn's disease and give them the proper care.

These results highlight the urgent requirement to focus on the psychological health of adolescents with Crohn's disease and to increase access to psychological therapy. Offering hope to Crohn's disease patients, lowering their negative emotions, and guaranteeing adequate behavioral coping mechanisms can considerably help with the management of the disease and probable regression, as described in another study [26].

### Focus on adaptive stress coping to promote positive experiences of the illness

The capacity of adolescents to manage stress successfully has a big impact on both their mental toughness and their problem-solving capability when they deal with a variety of stressors connected to school and sickness at different periods [27]. In this study, some adolescent patients learned adaptive coping techniques to cope with stress and adversity and cope better with the obstacles of social life and learning. Others, however, suffered from poor performance and behavioral burnout, which prevented them from growing personally. A study revealed that patients with Crohn's disease actively seek disease-related information after identifying stress associated with their condition, proactively acquire professional medical knowledge and health guidance, gain a thorough understanding of their illness, and decide their future actions while living with the disease [28].

It is essential to pay attention to the stress coping strategies used by adolescent patients with Crohn's disease and to motivate them to adopt adaptive coping strategies. Maintaining a positive view on life and learning are integral elements, as is actively seeking out social help in the form of knowledge and emotional support. These methods are crucial for ensure disease in remission and raising the general quality of life.

### Build a health care support system composed of multidisciplinary teams to optimize disease outcomes

Effective social support is essential for helping patients identify and manage their symptoms and for ensuring that they follow their treatment plan. Their capacity to look after themselves and practice health-promoting habits is subsequently improved [29]. For adolescents with Crohn's disease, getting the right medical treatment and care is important, but so is getting the support they need to preserve their mental health, promote physical growth, and get the nutrition they need to fend against the disease's progression. As a result, it is crucial to build a healthcare support system that consists of interdisciplinary teams with knowledge in psychology, medicine, nursing, nutrition, and other fields. Research and recommendations indicate that patients with Crohn's disease who are receiving nasal feeding need a multidisciplinary team that includes medical, nursing, and nutritional experts [30, 31]. With the help of such a team, patients can receive nutritional advice and specialist expertise, which will help them feel more capable of controlling their illness and promote its remission [19, 32, 33]. Furthermore, when adolescents with Crohn’s disease and their caregivers collaborate to improve the patients’ self-management skills and provide educational support, it can empower the patients to benefit from facing the challenges and pressures brought on by the disease. Their quality of life may be enhanced overall and unpleasant emotions may be lessened, which will aid in better disease management.

## Conclusion

Adolescents with Crohn's disease need to develop efficient coping mechanisms that can help them move from a passive to an active coping mode when under stress. This will help them better control their condition. Through the effective implementation of thorough assessments, diagnostic tests, and care coordination, nurses can significantly influence the treatment of IBD.Adolescents can be helped in navigating the physical and psychological obstacles of living with the major implications of Crohn's disease by developing a solid, trust-based healthcare relationship and through ongoing care throughout their journey of living with this disease.

## Data Availability

The datasets used and/or analysed during the current study available from the corresponding author on reasonable request.

## Abbreviations

CD: Crohn’s disease
IBD: Inflammatory Bowel Disease

## References

[1] Torres J, Mehandru S, Colombel JF (2017). Crohn’ disease. Lancet.

[2] Li GW, Ren JA (2017). Attaching importance to the study of the epidemiology of Crohn's disease in our country. Parenter Enter Nutr.

[3] Stelzig O, Sevecke K (2019). Stressbewältigung im Kindes- und Jugendalter .Coping with Stress During Childhood and Adolescence. Prax Kinderpsychol Kinderpsychiatr..

[4] Matsuoka K, Kobayashi T, Ueno F (2018). Evidence-based clinical practice guidelines for inflammatory bowel disease. J Gastroenterol.

[5] Li Z, Liu Y. [Research Methods of nursing][M].People's Health Publishing House, 2012.

[6] Consensus on the Diagnosis and Treatment of Inflammatory Bowel Disease (Guangzhou, 2012)].Wei Chang Bing Xue, 2012,17(12):763–781.

[7] Liu M (2019). The Application of Colaizzi's Seven Steps in the Analysis of phenomenology Research Data. Hu Li Xue Za Zhi.

[8] Fu H, Kaminga AC, Peng Y (2020). Associations between disease activity, social support and health-related quality of life in patients with inflammatory bowel diseases: the mediating role of psychological symptoms. BMC Gastroenterol.

[9] Finamore A, Peluso I, Cauli O (2020). Salivary Stress/Immunological Markers in Crohn's Disease and Ulcerative Colitis. Int J Mol Sci.

[10] Norton C, Czuber-Dochan W, Artom M (2017). Systematic review: interventions for abdominal pain management in inflammatory bowel disease. Aliment Pharmacol Ther.

[11] Larsson K, Lööf L, Nordin K (2017). Stress, coping and support needs of patients with ulcerative colitis or Crohn's disease: a qualitative descriptive study. J Clin Nurs.

[12] Lazarus RS, Folkman S (1984). Stress, Appraisal, and Coping[M].

[13] Folkman S (1997). Positive psychological states and coping with severe stress. Soc Sci Med.

[14] Rozich JJ, Holmer A, Singh S (2020). Effect of Lifestyle Factors on Outcomes in Patients With Inflammatory Bowel Diseases. Am J Gastroenterol.

[15] Gracie DJ, Irvine AJ, Sood R (2017). Effect of psychological therapy on disease activity, psychological comorbidity, and quality of life in inflammatory bowel disease: a systematic review and meta-analysis[J]. Lancet Gastroenterol Hepatol.

[16] Philippou A, Sehgal P, Ungaro RC (2022). High Levels of Psychological Resilience Are Associated With Decreased Anxiety in Inflammatory Bowel Disease. Inflamm Bowel Dis.

[17] Luo D, Lin Z, Bian QG, Wang MF (2018). Systematic evaluation of qualitative research on self-management experience in patients with inflammatory bowel disease. Chin J Nurs.

[18] Krauthammer A, Harel T, Zevit N (2020). Knowledge of disease and self-management of adolescents with inflammatory bowel diseases[J]. Acta Paediatr.

[19] Bisgaard TH, Allin KH, Keefer L (2022). Depression and anxiety in inflammatory bowel disease: epidemiology, mechanisms and treatment. Nat Rev Gastroenterol Hepatol.

[20] Kamp KJ, West P, Holmstrom A (2019). Systematic Review of Social Support on Psychological Symptoms and Self-Management Behaviors Among Adults With Inflammatory Bowel Disease. J Nurs Scholarsh.

[21] Kamp KJ, Luo Z, Holmstrom A (2019). Self-Management Through Social Support Among Emerging Adults With Inflammatory Bowel Disease[J]. Nurs Res.

[22] Carlsen K, Phan BL, Pittman N (2019). Coping Among Parents of Teens With Inflammatory Bowel Disease[J]. Gastroenterol Nurs.

[23] Jefferson K (2020). Living with Crohn's: A Personal Account. J Christ Nurs..

[24] Neuendorf R, Harding A, Stello N (2016). Depression and anxiety in patients with Inflammatory Bowel Disease: A systematic review. J Psychosom Res..

[25] Zhao YY, Gu J, Zhang YY (2018). Investigation and analysis of symptom burden in patients with inflammatory bowel disease. Chin J Modern Nurs.

[26] Lynch T, Spence D (2008). A qualitative study of youth living with Crohn disease. Gastroenterol Nurs..

[27] Gao X, Tang Y, Lei N (2021). Symptoms of anxiety/depression is associated with more aggressive inflammatory bowel disease. Sci Rep.

[28] Larsson K, Lööf L, Nordin K (2017). Stress, coping and support needs of patients with ulcerative colitis or Crohn's disease:a qualitative descriptive study[J]. Clin Nurs.

[29] Shang XC, Lin Z, Bian QG (2019). A Study on the Hope Level and Its Influencing Factors in Patients with Inflammatory Bowel Disease. Hu Li Yan Jiu.

[30] Nakase H, Uchino M, Shinzaki S (2021). Evidence-based clinical practice guidelines for inflammatory bowel disease 2020. J Gastroenterol..

[31] Lamb CA, Kennedy NA, Raine T (2019). British Society of Gastroenterology consensus guidelines on the management of inflammatory bowel disease in adults. Gut.

[32] Severo JS, da Silva Barros VJ (2021). Effects of glutamine supplementation on inflammatory bowel disease: A systematic review of clinical trials. Clin Nutr ESPEN.

[33] Gao X, Tang Y, Lei N (2021). Symptoms of anxiety/depression is associated with more aggressive inflammatory bowel disease. Sci Rep.

